# Ventral lengthening is preferable to combined ventral lengthening and dorsal plication for penile length in hypospadias with severe ventral curvature

**DOI:** 10.1038/s41598-026-38874-0

**Published:** 2026-02-16

**Authors:** Lizhe Hu, Hongbo Liu, Xiang Yan, Guangjie Chen, Wei Ru

**Affiliations:** Department of Urology, Children’s Hospital, Zhejiang University School of Medicine, National Clinical Research Center for Child Health, Hangzhou, China

**Keywords:** Hypospadias, Curvature correction, Urethral plate transection, Ventral lengthening, Dorsal plication, Penile length, Hypospadias, Urethra

## Abstract

This study aims to compare the effects of ventral lengthening (VL) versus ventral lengthening combined with dorsal plication (VL + DP) techniques on penile length in hypospadias. Clinical data were retrospectively collected from patients who underwent hypospadias repairs with urethral plate transection between March 2017 and June 2023. Patients were divided into VL group and VL + DP group based on the curvature correction techniques. Two novel parameters—penile lengthening ratio (PLR) and curvature-adjusted penile lengthening ratio (CPLR)—were introduced to evaluate the effectiveness of penile lengthening. A total of 103 male patients with hypospadias and severe ventral curvature were included. In patients with a curvature degree after degloving (CDAD) greater than 45°, the CPLR was significantly higher in the VL group compared to that in the VL + DP group (0.0101 ± 0.0028 vs. 0.0085 ± 0.0024, p = 0.026). Among patients with proximal hypospadias, the CPLR in the VL group was significantly higher than that in the VL + DP group (0.0106 ± 0.0024 vs. 0.0093 ± 0.0026, p = 0.041). In patients with CDAD > 45° or proximal hypospadias, VL demonstrated superior penile elongation compared to VL + DP.

## Introduction

Hypospadias is one of the most common congenital anomalies of the male genitalia, with a reported prevalence ranging from 1 in 300 to 1 in 200 live births^1^. Approximately 35% of patients with hypospadias present with significant ventral penile curvature. Ventral curvature results from multiple factors, including ventral skin deficiency, Dartos fascia constriction, hypoplastic urethral plate, and asymmetric corporal development^2–5^. The severity and combination of these factors dictate the choice of corrective approach. Current surgical options for residual curvature degree after degloving (CDAD) > 30° include ventral lengthening (VL) and dorsal plication (DP)^6^. VL encompasses urethral plate transection, ventral corporotomies, and ventral corporoplasty using flaps or grafts. The decision between VL and DP depends on the underlying etiology of curvature, the surgeon’s expertise, and the desired functional and cosmetic outcomes.

Penile length preservation is particularly crucial in cases of proximal hypospadias, where patients often experience psychological distress regarding genital appearance during adolescence. Given the strong correlation between penile length and adult sexual satisfaction, current guidelines emphasize preserving penile length through intraoperative strategies, particularly for these high-risk populations^7–9^. Curvature correction remains a pivotal aspect of hypospadias repair, significantly influencing both surgical success and postoperative quality of life. Although advances have been made in correction strategies, comparative evaluations of outcomes across techniques remain limited.

In this single-center retrospective study, we aim to compare the effects of VL and VL + DP techniques on penile length preservation in patients with severe ventral curvature. Specifically, we focus on evaluating how these techniques perform in terms of penile lengthening, taking into account varying degrees of curvature and the type of hypospadias, to guide evidence-based decisions in the selection of optimal surgical strategies.

## Materials and methods

### Study design and patients

A retrospective study was conducted among pediatric patients who underwent primary hypospadias repair with curvature correction between March 2017 and June 2023 at a single tertiary center. Ethical approval was obtained from the Institutional Review Board of the Children’s Hospital, Zhejiang University School of Medicine (2024-IRB-0262-P-01), and all procedures were conducted in accordance with the principles of the Declaration of Helsinki. Informed consent was obtained from legal guardians prior to inclusion.

### Inclusion and exclusion criteria

Patients were eligible if they met the following criteria: (1) diagnosis of hypospadias with a CDAD > 30°; (2) underwent primary hypospadias repair with urethral plate transection; (3) had a minimum follow-up duration of 12 months. Patients with incomplete follow-up data or previous hypospadias surgeries were excluded.

### Interoperative measurements

**Fig. 1:**
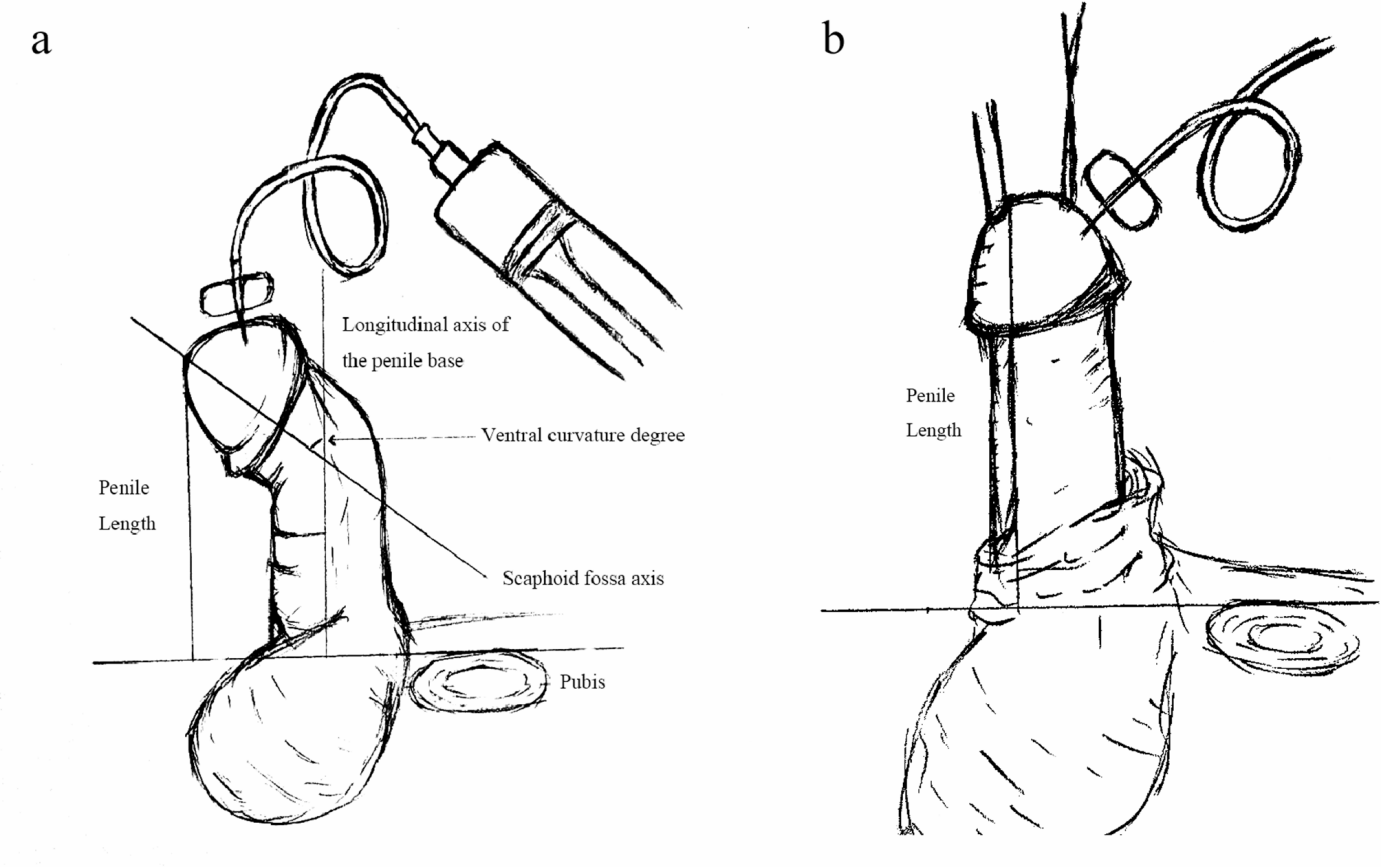
Assessment of preoperative and postoperative penile lengths. (**a**) Measurement of the ventral curvature degree and penile length after degloving, with the penis fully stretched from the pubic bone to the glans tip. (**b**) Measurement of penile length after curvature correction, including the evaluation of penile length and curvature correction post-straightening. Ventral curvature degrees were evaluated using a protractor during artificial erection testing. The primary outcome measures included penile lengthening and curvature correction outcomes.

According to Orkiszewski’s classification of hypospadias^10^, the division of the corpus spongiosum was examined relative to the shaft of the penis and the upper pubis. Hypospadias above the pubis was categorized as penile (distal), while below was proximal. Penile length was measured intraoperatively at two time points^11^. Measurements followed a standardized protocol with the penis fully stretched. The initial measurement was taken prior to curvature correction, while the second measurement was carried out immediately after the correction procedure was completed. The vertical distance from the superior border of pubic bone to the glans tip was recorded as penile length (Fig. 1). Ventral curvature degrees were assessed using a protractor during artificial erection testing (Fig. 1a).

### Curvature correction strategies

Patients were categorized into two groups based on the curvature correction strategies employed:

VL Group: This approach involved urethral plate transection and ventral corporotomies / corporoplasty to achieve ventral lengthening. Ventral corporotomies were performed via three or more transverse incisions along the concave aspect of the tunica albuginea. By contrast, ventral corporoplasty involved a deep transverse incision through the concave aspect of the tunica albuginea, followed by augmentation of the corporal defect with either a pedicled urethral plate flap^12^ or a vascularized Buck’s fascia flap.

VL + DP Group: In addition to the VL techniques, dorsal plication was performed by longitudinal incision of Buck fascia to expose the underlying tunica albuginea, followed by one or two 5 − 0 polypropylene stitch plication of the midline septum of the corpora.

A final curvature < 10° was considered to indicate successful curvature correction.

### Assessment of penile lengthening efficacy

Penile Lengthening Ratio (PLR): Ratio of penile lengthening length to postoperative penile length.

$$\:\mathrm{P}\mathrm{L}\mathrm{R}=\frac{Postoperative\:Penile\:Length-Preoperative\:Penile\:Length}{Postoperative\:Penile\:Length}\times\:100\%$$

Curvature-Adjusted Penile Lengthening Ratio (CPLR): PLR normalized by CDAD.

$$\:\mathrm{C}\mathrm{P}\mathrm{L}\mathrm{R}=\frac{PLR}{CDAD}$$

### Sample size calculation

This study is a retrospective study. Group 1 is the VL group, and Group 2 is the VL + DP group. PLR is the outcome measure. Based on the results of the pilot study, the allowable error is 0.008, and the standard deviation is 0.01. With a two-sided α of 0.05 and a power of 0.9, according to the sample size calculation formula, $\:n=\:\frac{{({Z}_{\alpha\:}\:+\:{Z}_{\beta\:})}^{2}\:\times\:\:2{\sigma\:}^{2}}{{\delta\:}^{2}}$, *n* = 32.8 is required. The sample size ratio between Group 1 and Group 2 is 1:1, with 33 cases in each group. Considering a 15% dropout and refusal rate, at least 39 cases are needed in Group 1 and 39 cases in Group 2, resulting in a total sample size of 78.

### Follow-up

Postoperative follow-up was conducted via outpatient visits, and online platforms (WeChat©, Tencent Holdings). To more objectively assess the cosmetic appearance of patients postoperatively, we utilized the HOPE (Hypospadias Objective Penile Evaluation) scoring system^13^. The postoperative complications recorded included recurrent curvature, fistula, urethral stricture, meatal stenosis, urethral diverticulum, urethral dehiscence, glans dehiscence, and erectile dysfunction. Erectile function was assessed by parental report of penile rigidity during spontaneous erections. Meanwhile, lateral penile photographs were obtained and independently reviewed by the surgeon to evaluate for recurrent curvature. Recurrent curvature was diagnosed when the curvature degree ≥ 10°.

### Statistical analysis

Continuous variables were tested for normality using the Shapiro-Wilk test. Normally distributed variables were expressed as mean ± standard deviation (M ± SD) and compared using the independent samples t-test. Non-normally distributed variables were reported as median (interquartile range) and analyzed using the Mann-Whitney U test. Categorical variables were presented as frequencies and percentages, with group differences assessed using the Chi-Square or Fisher’s exact test. Multivariable linear regression models were applied to evaluate associations between curvature correction strategies and penile lengthening outcomes. A *p*-value < 0.05 was considered statistically significant. Statistical analyses were performed using R 4.4.1 and GraphPad Prism 9.0.

## Results

### Patient characteristics

A total of 119 patients met the inclusion criteria. Sixteen (13.4%) were lost to follow-up, resulting in 103 patients being included in the final analysis. The median age was 1.583 [1.000, 2.750] years, and the median follow-up duration was 2.247 [1.463, 3.707] years. According to Orkiszewski’s classification of hypospadias[10], the location of the corpus spongiosum division was penile in 36 cases (34.95%) and proximal in 67 cases (65.05%). The median preoperative curvature was 70.0°[60.0, 90.0°], reducing to 60.0° [45.0, 90.0°] after degloving (CDAD).

Among 103 patients, 44 (42.72%) underwent VL and 59 (57.28%) underwent VL + DP. Baseline characteristics were comparable between groups, including median age, hypospadias classification, preoperative curvature, and CDAD (*p* > 0.001). There were no significant differences in PLR or CPLR between the VL and VL + DP groups. However, the follow-up was significantly longer in VL + DP group compared to the VL group (3.42 [2.48, 4.15] vs. 1.40 [0.97, 1.89] years, *p* < 0.001).

All 103 cases underwent urethral plate transection as the foundational surgical step. For further curvature correction, distinct intergroup patterns adopted (Table 1). Technical outcomes revealed 2/27 (7.41%) corporoplasty cases required supplemental dorsal plication contrasting sharply with corporotomy cases where 10/14 (71.43%) needed combined plication (χ^2^ = 32.15, *p* < 0.001).

**Table 1:** Clinical Characteristics.

Clinical Characteristics	VL (*n* = 44)	VL + DP (*n* = 59)	*p*-value
Age (y)	1.417 (0.771, 2.938)	1.833 (1.000, 2.667)	0.397
Follow-up (y)	1.397 (0.966, 1.890)	3.422(2.477, 4.145)	< 0.001
Hypospadias classification			
Distal	19 (43.18%)	17 (28.81%)	0.130
Proximal	25 (56.82%)	42 (71.19%)	
Preoperative curvature degree (°)	60 (60, 90)	80 (60, 90)	0.455
CDAD (°)	60 (45, 70)	60 (45, 90)	0.396
PLR	0.5874 ± 0.2783	0.5754 ± 0.2105	0.724
CPLR	0.0098 ± 0.0022	0.0091 ± 0.0024	0.133
Ventral correction techniques			
Urethral plate transection	44 (100%)	59 (100%)	1.000
Corporotomies	4 (9.09%)	10 (16.95%)	< 0.001
Corporoplasty	25 (56.82%)	2 (3.39%)	< 0.001
Dorsal plication	0	59 (100%)	< 0.001
Urethroplasty techniques			
Duckett	31 (70.45%)	29 (49.15%)	0.855
Duckett + Duplay	7 (15.91%)	13 (22.03%)	0.114
Koyanagi	1 (2.27%)	4 (6.78%)	0.206
Two-stage Duckett	3 (6.82%)	11 (18.64%)	0.007
Two-stage Byars	2 (4.55%)	2 (3.39%)	1.000
Complication	12 (27.27%)	17 (28.81%)	0.294
Fistula	7 (15.91%)	11 (18.64%)	0.317
Diverticulum	3 (6.82%)	5 (8.47%)	0.619
Urethral stricture	0	2 (3.39%)	0.333
Meatal stenosis	3 (6.82%)	2 (3.39%)	1.000
Glans split	0	1 (1.69%)	1.000

Continuous variables are expressed as median (interquartile range), and categorical variables are presented as n (%).

VL, ventral lengthening; VL + DP, ventral lengthening + dorsal plication; CDAD, curvature degree after degloving; PLR, penile lengthening ratio; CPLR, curvature-adjusted penile lengthening ratio.

The overall complication rate was 28.16%, with no significant difference between groups (*p* = 0.294) (Table 1). No recurrent curvature was observed.

### Analysis of factors influencing penile lengthening outcomes

Univariate regression analysis demonstrated significant associations between PLR and CDAD (coefficient = 0.0072, 95% CI 0.0059–0.0084, *p* < 0.001) as well as proximal hypospadias classification (coefficient = 0.323, 95% CI 0.247–0.399, *p* < 0.001). In contrast, neither age (*p* = 0.736) nor curvature correction strategy (VL vs. VL + DP, *p* = 0.883) showed significant associations with PLR.

**Fig. 2:**
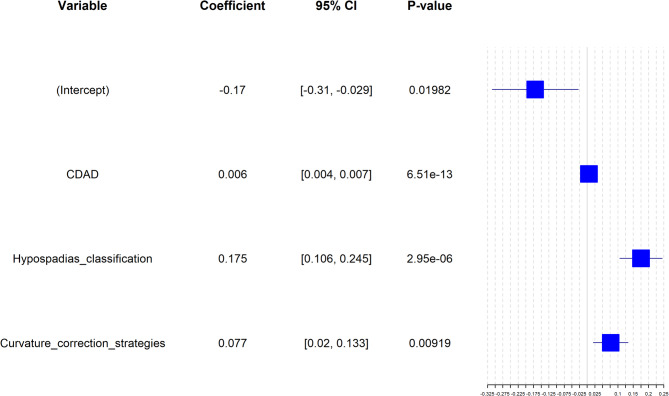
Linear regression analysis of penile lengthening ratio (PLR). A linear regression analysis with PLR as the dependent variable and baseline clinical characteristics as independent variables, revealed that patients with more severe curvature degree after degloving (CDAD), proximal type of hypospadias, and no dorsal shortening had better penile lengthening outcomes.

The potential variables were further assessed in a multivariable linear regression model. The multivariable analysis revealed that higher CDAD (coefficient = 0.0056, 95% CI 0.0043–0.0069, *p* < 0.001), proximal hypospadias (coefficient = 0.175, 95% CI 0.106–0.245, *p* < 0.001) and venture correction strategy with VL alone (coefficient = 0.077, 95% CI 0.020–0.133, *p* = 0.009) were associated with higher PLR (Fig. 2).

Model validation confirmed that all variance inflation factors < 2.0, and sensitivity analyses yielding consistent findings across alternative model specifications.

### Penile lengthening efficacy between subgroups

**Fig. 3:**
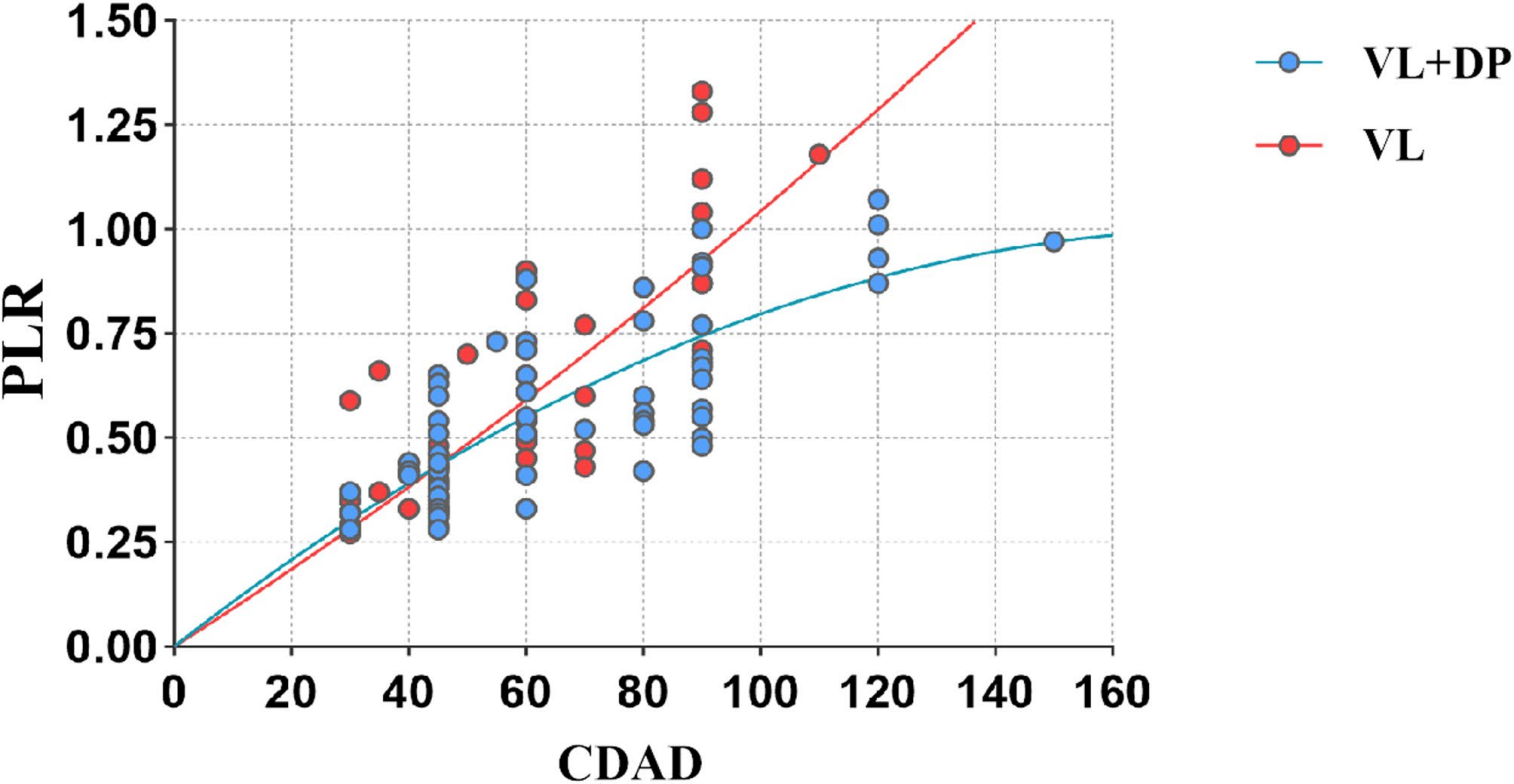
Scatter plot of ventral curvature degree after degloving (CDAD) and curvature-adjusted penile lengthening ratio (CPLR). A scatter plot was created with Curvature degree after degloving (CDAD) on the horizontal axis and the Penile lengthening ratio (PLR) on the vertical axis. The fitted quadratic curves intersect at 45°, and thus a cut-off value of 45° was selected. VL, ventral lengthening; VL + DP, ventral lengthening + dorsal plication.

**Fig. 4:**
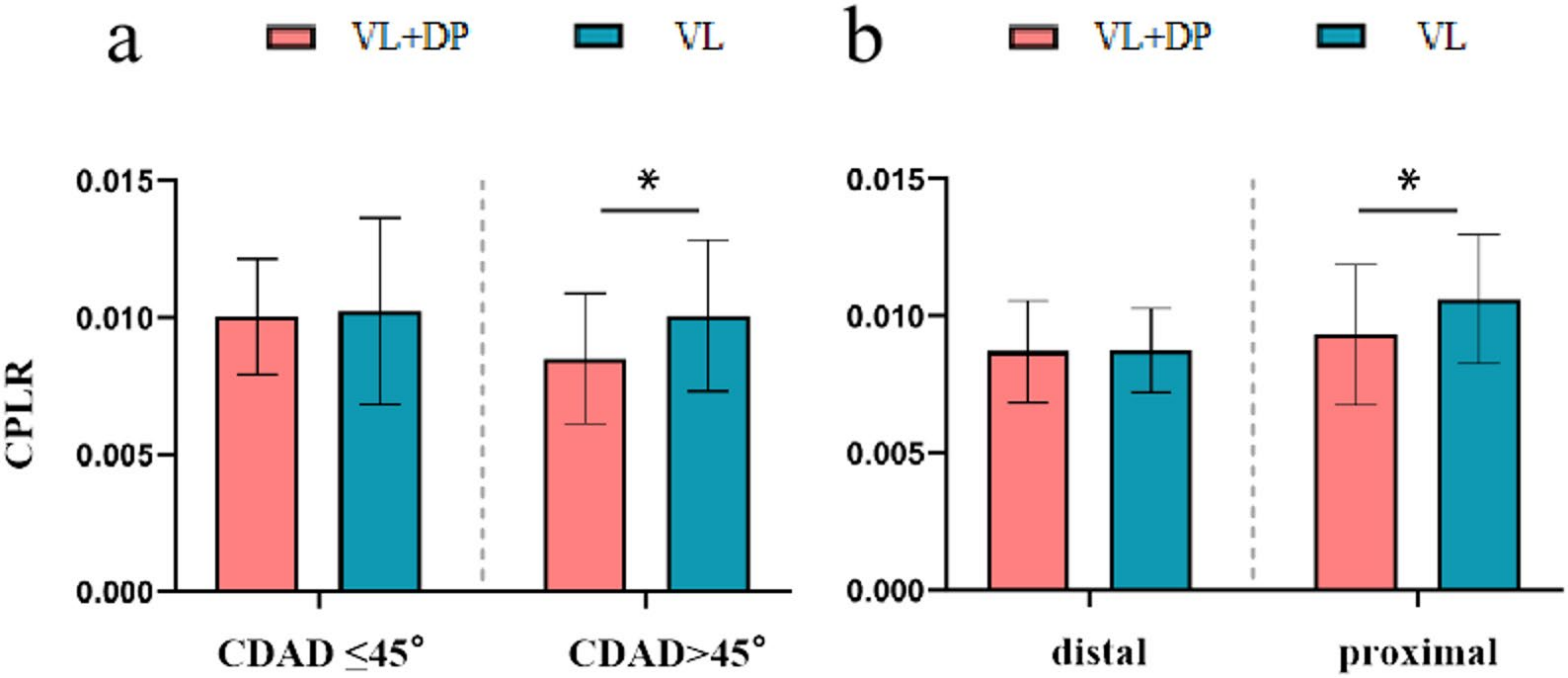
Grouped bar chart of curvature-adjusted penile lengthening ratio (CPLR), stratified by CDAD and hypospadias classification. Grouped bar chart depicting the curvature-adjusted penile lengthening ratio (CPLR) on the vertical axis. Groups were stratified by CDAD ≤ 45°vs CDAD > 45° (**a**), and by hypospadias classification (**b**). *p < 0.05. VL, ventral lengthening; VL + DP, ventral lengthening + dorsal plication.

A scatter plot was generated to illustrate the relationship between the PLR and CDAD, followed by polynomial fitting (Fig. 3). The R² value was 0.951 for the VL group and 0.950 for the VL + DP group, indicating a strong curve fit. The figure demonstrates that CDAD significantly affects penile lengthening outcomes. In the fitted curves, we found that when CDAD < 45°, the two curves roughly overlap, and when CDAD > 45°, the two curves gradually separate. To further investigate this, we introduced the parameter CPLR. Based on the scatter plot, a cut-off value of 45° was selected, dividing patients into two groups: CDAD > 45° and CDAD ≤ 45°. Subgroup analysis revealed that among patients with CDAD > 45°, the VL group exhibited a significantly higher CPLR compared to that in the VL + DP group (0.0101 ± 0.0028 vs. 0.0085 ± 0.0024, *p* = 0.026) (Fig. 4a). Similarly, in proximal hypospadias cases, VL resulted in significantly greater penile elongation (0.0106 ± 0.0024 vs. 0.0093 ± 0.0026, *p* = 0.041) **(**Fig. 4b).

### Postoperative evaluation of cosmetic appearance

Out of the 103 cases, 76 underwent HOPE scoring. Among them, 34 patients were in the VL group (77.27%), and 42 patients were in the VL + DP group (71.19%). The mean HOPE scores for the two groups were as follows: the VL group scored 9.705 ± 0.4570, and the VL + DP group scored 9.655 ± 0.4393. The difference between the two groups was not statistically significant ( *p* = 0.5701). These results indicate that there was no significant difference in the cosmetic outcomes between the two surgical approaches as measured by the HOPE scoring system.

## Discussion

This study compared two curvature correction strategies, VL and VL + DP, and found that CDAD, hypospadias type, and the use of DP were significantly associated with penile lengthening outcomes. VL demonstrated superior results compared to VL + DP, particularly in patients with CDAD > 45° and proximal hypospadias. These findings provide evidence-based guidance for surgical decision-making in patients with CDAD > 45° and proximal hypospadias.

Previous studies, such as Zhou et al. have demonstrated that urethral plate transection effectively elongated the penis in both distal and proximal hypospadias, but they did not explore the impact of other different curvature correction techniques^14^. Our use of normalized ratios (CPLR) allowed for more accurate comparisons across varying baseline characteristics. Polynomial regression identified 45° as a critical threshold, confirming that VL achieved significantly greater lengthening than VL + DP in patients exceeding this curvature (*p* = 0.028).

The follow-up duration was significantly longer in the VL + DP group. However, sensitivity analyses adjusting for follow-up time demonstrated consistent treatment effects, suggesting that the observed complications are unlikely to be solely attributable to follow-up duration. The penile length was measured intraoperatively, ensuring that the assessment of penile lengthening efficacy remained unaffected by follow-up duration. In practice, the VL strategies not only achieve superior penile lengthening results but also show no disadvantages in terms of short-term complications.

VL and DP are the main surgical techniques currently used for penile curvature correction in clinical practice. DP is favored by some surgeons due to its simplicity and minimal invasiveness. DP poses risks, such as potential damage to the dorsal neurovascular bundle, which may lead to complications such as penile pain during erection and suture-related issues. Additionally, in cases of severe curvature, DP is associated with a higher recurrence rate. Acimi et al.^15–17^ found that the main cause of curvature in proximal hypospadias is fibrotic tissue on the ventral side of the penis and hypoplastic ventral tissues, suggesting that DP may not be the ideal correction technique. Recent meta-analyses have demonstrated that VL techniques are associated with significantly lower recurrence rates of curvature compared to DP^18^. Snodgrass^19^ recommended VL instead of DP for residual curvature greater than 30° post-degloving, especially when DP may significantly reduce penile length. In a retrospective study of 102 patients who underwent DP for ventral curvature, Greenfeld et al.^20^ found that the incidence of penile shortening increased with greater curvature severity. Specifically, the shortening rate was approximately 1% for curvatures ≤ 45°, about 2% for curvatures between 45° and 60°, and as high as 4% for curvatures > 60°. This suggests that DP has a more pronounced negative impact on penile length in cases of severe curvature and help explain the superior penile lengthening outcomes observed with VL alone compared to VL + DP.

At our center, various curvature correction techniques were employed based on the patient’s penile condition. A particularly noteworthy technical finding in our study was that corporoplasty cases rarely required supplemental dorsal plication (7.41%), in stark contrast to corporotomy cases, where 71.4% needed combined plication (χ² = 32.15, *p* < 0.001). This finding aligns with our previous report^12^, which highlighted that corporoplasty, by performing a deep transverse incision at the point of maximum curvature, allows for more precise and complete ventral lengthening of the tunica albuginea. In contrast, corporotomy, whether involving single or multiple incisions, often fails to achieve fully straightening due to concerns about preserving corporal integrity. This may explain why the majority of corporotomy cases still required additional dorsal plication to fully correct residual curvature.

Recurrence of curvature is often related to uneven ventral corpus spongiosum development or a mismatch in urethral growth after reconstruction^21,22^. Unlike traditional graft or flap materials such as dermis, small intestinal submucosa, tunica vaginalis, or dura mater, we preferred the urethral plate flap for corpus spongiosum reconstruction due to its high anatomical similarity to the corpus cavernosum. Snodgrass^23^ reported that the urethral plate in hypospadias patients develops normally, including the tunica albuginea and corpus spongiosum. When the UPF is flipped over and patched onto the corporal defect, the albuginea lies externally, and the stratum spongiosum internally, mimicking normal tissue structure. Furthermore, the dense structure of the tunica albuginea enables secure suturing and reduces the risk of bleeding from the corpus cavernosum. When the urethral plate was too thin to obtain a suitable urethral plate flap, the Buck’s fascia flap was our second choice for corpus spongiosum reconstruction. However, potential complications such as erectile dysfunction and scarring following tunica albuginea incisions remain a concern due to the lack of adequate long-term follow-up data^24^.

Research on the relationship between hypospadias type and penile lengthening is limited. Nearly all patients with proximal hypospadias exhibit a certain degree of ventral curvature^25^. In this study, we found that patients with proximal hypospadias experienced significantly greater penile lengthening in VL group compared to those in VL + DP group. We believe that VL is a superior option for patients with proximal hypospadias.

Previous studies have reported complication rates of 50–80% in severe hypospadias cases^6,26,27^. In this study, the overall complication rate was 28.16%, which is lower than previously reported in the literature. Additionally, we did not observe any cases of recurrent curvature. All postoperative complications were effectively addressed and resolved through one or two reoperations. No significant long-term issues arose following the corrective surgeries.

This study has several limitations. Firstly, its small sample size and retrospective, single-center design may introduce selection biases in surgical technique choice. Additionally, the short follow-up duration limits our ability to assess long-term outcomes, particularly in adolescence when concerns about penile appearance and function become more prominent. A longer follow-up period, especially extending into adolescence with repeated penile length measurements, would provide a more comprehensive understanding of the procedure’s long-term efficacy, safety, and potential late complications, such as urethral stricture. Finally, multi-center studies with larger sample sizes and standardized techniques are essential to validate these findings and further explore the long-term outcomes of different curvature correction strategies.

## Conclusion

For patients with CDAD > 45° and proximal hypospadias, the VL strategy significantly improves penile lengthening compared to the combined VL + DP approach. These findings provide valuable insights to guide clinicians in optimizing surgical strategy selection in hypospadias repairs, highlighting the importance of considering curvature severity and hypospadias type in determining the best treatment approach.

## Data Availability

Data are available from the corresponding author on reasonable request.

## Abbreviations

CDAD: Curvature degree after degloving
DP: Dorsal plication
VL: Ventral lengthening
PLL: Penile lengthening length
PL: Penile length
VL + DP: Ventral lengthening + dorsal plication
PLR: Penile lengthening ratio
CPLR: Curvature-adjusted penile lengthening ratio
M: Mean
SD: Standard deviation
